# Saturated Fatty Acid Chain Length and Risk of Cardiovascular Disease: A Systematic Review

**DOI:** 10.3390/nu15010030

**Published:** 2022-12-21

**Authors:** Monica Perna, Susan Hewlings

**Affiliations:** 1 Fresenius Kabi 3 Corporate Dr, Lake Zurich, IL 60047, USA; 2 Nutrasoource 120 Research La, Guelph, ON N1G 0B4, Canada

**Keywords:** saturated fatty acid, cardiovascular disease, cholesterol, medium-chain fats, saturated fat chain length

## Abstract

The purpose of this systematic review was to evaluate the impact of saturated fatty acid chain lengths on the development of cardiovascular disease (CVD). The importance of replacement macronutrients is also discussed. PubMed, CINAHL, and Cochrane library were searched for relevant prospective cohort studies that measured SFA chain length via diet analysis through October of 2020. A second updated PubMed search was conducted from October 2020 to 7 August 2022. Five prospective cohort studies were added. All studies used food frequency questionnaires to assess dietary intake. For all five added studies, the main sources of saturated fat were palmitic and steric acid from meat and cheese. Most studies discovered an association with increased risk of CVD and long-chain saturated fatty acid intake, as well as a neutral (potentially beneficial) association with short- and medium-chain saturated fatty acids. Isocaloric substitutions were associated with a higher risk for CVD when saturated fats were replaced with refined carbohydrates and protein from meat, but a reduced or neutral impact when relaced with plant-based protein, unsaturated fat, or complex carbohydrates. When examining the impact of diet on CVD risk, it is critical to consider the macronutrient replacing saturated fat as well as the saturated fat chain length, whole foods, and diet patterns on CVD risk. The studies included in this review suggest that LCSFA (C12–18) may increase the risk for CVD development, while SCFA and MCFA (C4–-C10) may be more beneficial or neutral.

## 1. Introduction

The leading cause of morbidity and mortality worldwide is cardiovascular disease (CVD) [1,2,3]. In the United States, the cost to treat CVD is over USD 200 billion per year [1]. Therefore, preventative measures including dietary modification have been a focus of the American Heart Association (AHA) and the American College of Cardiology (ACC) public health recommendations for decades [1,2]. Development of CVD has been linked to saturated fat intake due to its proposed impact on serum total cholesterol levels [4,5]. Specifically, it has long been suggested that dietary saturated fat consumption increases low-density lipoprotein (LDL), which is a cause of CVD [6,7,8,9,10]. This idea is known as the cholesterol hypothesis [8]. Since 1977, dietary guidelines have focused on reducing saturated fat intake to 10% or less of total calorie needs per day [2,6,11,12,13,14,15]. It is also widely accepted that polyunsaturated fatty acids (PUFA) reduce LDL levels and therefore are generally recommended instead of saturated fat [2,3,14,16,17]. The current accepted dietary guidelines to reduce CVD risk include decreasing saturated fat intake, consuming fish or plant sources of PUFA, and avoiding trans fatty acids (TFA) [18,19].

In contrast to the long-standing dogma, recent research has questioned the recommendation to reduce total saturated fat [6,7,13,19]. It has been suggested that dietary management may involve more than just decreasing total saturated fat to lower LDL levels [14]. One recommendation is to take into account the influence of diet on other lipoproteins beyond LDL [19]. Just as LDL may predict an increased risk for CVD, high-density lipoprotein (HDL) has been associated with a lower risk [20]. Mensink et al. suggested that the total cholesterol to high-density lipoprotein cholesterol (total:HDL) ratio is a more significant indicator than just LDL [5]. Thus, how various foods and dietary patterns influence the entire lipid profile is of importance. This makes what one replaces saturated fat with when one makes dietary changes of great significance for CVD risk reduction [5,6,7,16,17,20]

As part of this recent scrutiny, the idea that chain length of individual SFA should be evaluated for their unique lipid-modulating properties has been proposed [6,21,22]. SFA can be categorized into three different groups: short-chain fatty acids (SCFA), medium-chain fatty acids (MCFA), and long-chain saturated fatty acids (LCSFA) [6]. SCFA are less than six carbons in length and are limited in dietary sources [6,23,24]. The majority of SCFA are produced in the colon as biproducts of fiber fermentation [6,23]. Although this paper does not focus on dietary fiber, it is important to note that SCFA have been linked to reduced plasma cholesterol levels [23,25,26]. MCFA are 6–12 carbons in length [6,24], with food sources including palm kernel oil, coconut oil, and dairy products [6,22]. Finally, LCSFA are greater than 12 carbons in length [6,24], and major food sources are diary, beef, palm oil, and lard [6,22]. Current guidelines do not differentiate chain length of saturated fat sources, despite awareness that different chain lengths have different health effects.

Therefore, the aim of this review was to examine the unique effects of the different chain lengths of individual SFA on the development of CVD in adults.

## 2. Materials and Methods

The current systematic review and meta-analysis was conducted and reported in accordance with the PRISMA statement checklist [27]. The eligibility criteria included prospective cohort studies of adults with an evaluation period of at least two years. This study design was chosen due to the chronic nature and disease latency of the development of CVD as it relates to diet patterns. Only studies that measured usual oral intake and analyzed chain length of dietary intake of SFA were included.

**Figure 1:**
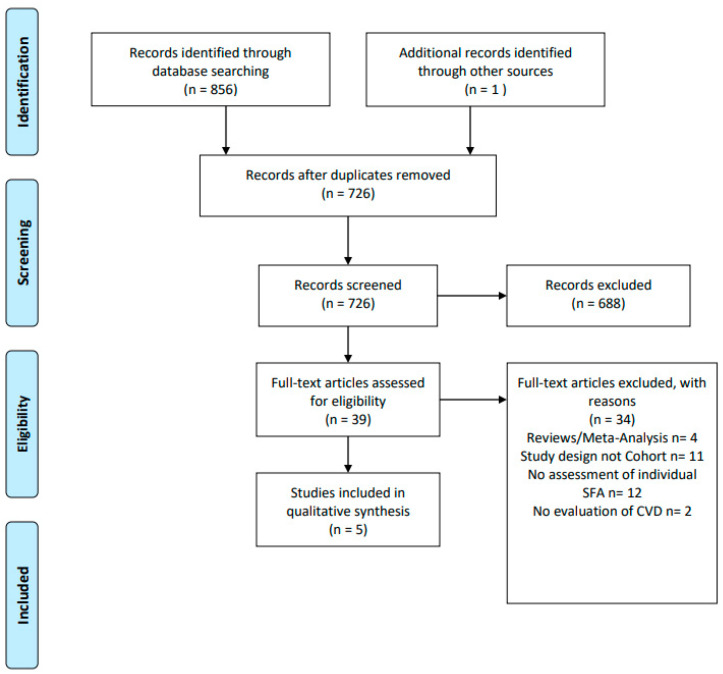
PRISMA 2022 flow diagram.

PubMed, CINAHL, and Cochrane library were originally searched for relevant literature in October of 2020. A second PubMed search was conducted from October 2020 to 7 August 2022 in order to obtain any additional qualifying literature. Search terms included cardiovascular disease, myocardial infarction, ischemic heart disease, atherosclerosis, dyslipidemia, cholesterol, saturated fatty acids, palmitic acid, stearic acid, lauric acid, myristic acid, MCT, LCT, cohort studies, and observational. No date restriction was imposed on this literature search, and only studies in the English language were included. Reference lists were also reviewed as indicated for possible qualifying studies. This resulted in 856 studies, of which 130 duplicates were removed, leaving 726 studies. On the basis of title and abstract screening, 688 studies were eliminated. A full text review was completed on the remaining 39 studies, resulting in the exclusion of 34, leaving 5 studies fitting the eligibility criteria. To assess bias, the National Institution of Health (NIH) Study Quality Assessment for Cohort and Cross-Sectional Studies was applied to all research papers included in this review [28]. See Figure 1 for search details.

## 3. Results

### 3.1. Bias Assessment

Use of cohort studies allows for improved statistical power often needed for the evaluation of a disease/nutrient relationships [13]. However, cohort studies are inherently biased due to the reliance of self-reporting of dietary patterns and the risk for under- or overestimations [29].

Only one of the studies evaluated in this review noted “rate of follow-up” [30]. Three of the studies only obtained dietary data at the beginning of the study period as baseline measures [30,31,32]. Of these three studies, the lowest mean follow-up time was 12.2 years [31]. It is reasonable to suspect a person’s diet could change over the course of that time span. To address this, along with reverse causation, the researchers of these studies also conducted additional analyses [30,31,32]. One study reported stopping the collection of dietary data when a diagnosis of stroke, diabetes, or cancer was made to reduce the risk for reversal causation bias [33]. All five studies included in this review corrected for multiple confounding variables, but it is impossible to eliminate all confounding variables [22,30,31,32,33]. Finally, only two studies noted that physicians/assistants were blinded when used to clarify medical records [22,33]. The NIH Study Quality Assessment Tool for Observational Cohort and Cross-Sectional Studies was completed on all research included in this review [28]. On the basis of all the above information, the risk for overall bias was low to unclear (see Table 1).

### 3.2. Study Characteristics

See Table 2 for a summary of the included studies. All included studies were consistent with their measurement of assessment outcomes, which were completed via participant report, next of kin, electronic medical records, and death records [22,30,31,32,33]. Study physicians and/or medical assistants validated diagnosis using industry standards of practice in three of the studies [22,32,33], but only two reported this practice was conducted in a blinded fashion [22,33]. One study referenced the rate of follow-up as being close to 100% [30], while the other studies did not report follow-up rates [22,31,32,33].

Multivariant analysis was conducted in all studies for several confounding factors including, but not limited to, smoking status, education level, physical activity, alcohol use, anthropometrics, and cholesterol level [22,30,31,32,33].

In three studies, the years of follow-up per person were calculated using the date of completion of the baseline questionnaire until the date of either CVD diagnosis, death, or conclusion of the follow-up period (whichever came first) [22,31,33]. The other two studies did not identify how the years of follow-up per person was calculated in their methodology [30,32]. Zong et al. included both probable and confirmed cases of CVD, and a sensitivity analysis was conducted by excluding probable fatal cases [33]. Cohorts were divided into quintiles, and hazard ratio (HR) was calculated with a 95% confidence interval (95% CI) for CVD events and SFA intake along with a Cox proportional hazards regression model for variables [30,31,32,33]. One study calculated relative risk ratios and 95% CI instead of hazard ratios [22]. In one study, two cohorts were analyzed individually first, and then the results were pooled together [30].

Dietary intake was obtained using food frequency questionnaires (FFQ) that were verified by either 24 h recalls or food records for four studies included in this review [22,31,32,33]. However, in one study, the FFQ was not validated to analyze saturated fat (EPIC-Norfolk) [30]. Macronutrients were calculated as a percentage of total calories, and individual SFA were analyzed using food analysis software [22,30,31,32,33]. Only two studies obtained repeat measures of diet intake during the study, in which cumulative averages were calculated to better represent habitual intake [22,33]. The three studies that did not obtain a repeat measure of diet intake conducted repeat analysis of the first 5–8 years independently to see if the relationship of the baseline data changed with time [30,31,32].

To analyze individual SFA, butyric acid, caproic acid, caprylic acid, and capric acid (C4-C10) were analyzed together as short- to medium-chain fatty acids (FA) due to very low intake and similar food sources [22,30,31,32,33]. Two studies had low intake for lauric acid (C12) and shared food sources with myristic acid (C14) [22,30]. Therefore, one study evaluated them individually and together [22,30], while the other study only looked at them together [22]. In three studies, pentadecylic acid (C15) and margaric acid (C17) were analyzed together due to low intake and shared foods [30,32]. In one of these studies, however, one of the two cohorts included had no data for C17 (EPIC-Denmark cohort), so only C15 was analyzed independently [30]. Palmitic acid (C16) and stearic acid (C18) were evaluated individually in all studies [22,30,33]. C12-C18 were analyzed together as a group in three studies [22,30,31,32,33]. Isocaloric substitution analysis was completed in all five studies for PUFA [22,30,31,32,33]. Four studies analyzed substitution of SFA with cis MUFA, vegetable protein sources, meat protein sources, and carbohydrates (CHO) [30,31,32,33]. The type of CHO substitutions was evaluated in two studies [31,32]. In one study, TFA intake was only provided for ruminant sources for one of the two cohorts analyzed (Danish Cohort) and therefore was left out of the analysis [30].

### 3.3. Diet/Lifestyle Characteristics

Baseline measures recorded overall saturated fat intake at greater than 10% for all studies [22,30,31,32,33]. However, the studies that obtained repeat FFQ showed an overall reduction in intake over time, with some just under 10% towards the end of the study periods [22,33]. For all five studies, participants with higher saturated fat intake were less active and consumed higher amounts of cis MUFA [22,30,31,32,33]. In four out of five studies, higher intake of saturated fat was associated with smoking [22,30,31,32], greater intake of TFA [30,31,32,33], lower intake of CHO [30,31,32,33], lower intake of fiber [22,30,31,32], and lower intake of alcohol [22,30,31,33].

### 3.4. Saturated Fat, SFA Chain Length, and Food Sources

Two studies analyzed the risk for CVD and intake of total saturated fat [31,32,33]. One found no association (HR5en%: 1.13; 95% CI:0.94, 1.36) [32] and the other found a significantly lower risk of ischemic heart disease (IHD) (HR5en%: 0.89; 95% CI: 0.74, 0.93) [31]. For all five studies, the main source of SFAs were C16 and C18 [22,30,31,32,33], with three studies specifically noting food sources primarily from meat and cheese [22,31,32].

In three studies, intake of C4-C10 had no link for CVD after all variables were analyzed [22,32,33]. However, Praagman et al. found a reduction for risk of IHD associated with intake of C4-C10 (HR: 0.93; 95% CI 0.89, 0.99) [31]. In the EPIC-Norfolk/Denmark study, an inverse association with C4-C10 intake was observed within the Denmark cohort. However, this link was weak and non-significant in the Norfolk Cohort. The pooled results indicated a non-linear inverse or neutral association [30].

Two studies found C12 had no significant association with CVD [31,32]. However, two other studies found a higher risk associated with C12 intake [22,33], although in one of these two studies, this association was significantly decreased with adjustments for confounding variables [22]. In the EPIC-Norfolk/Denmark study, there was a decreased risk for MI correlated with C12 intake in the Denmark cohort; however, the pooled results showed an inverse or neutral relationship [30].

Two studies found a higher risk for CVD associated with C14 intake [22,33], with one study noting that the results were significantly decreased with adjustments for confounding variables [22]. The other three studies found an inverse relationship or reduced risk [30,31,32].

Combined results for C12 and C14 were examined in two studies due to shared food sources, which found an inverse relationship to MI risk [30] or a non-significant impact after correction for confounding variables [22].

Only three studies evaluated the combination of C15 and C17, with one finding a notably lower risk (HR: 0.91; 95% CI: 0.83, 0.99) for IHD [31]. Another found the total of C15 and C17 had an inverse relationship to MI risk [30], while the last one found no significant association [32].

Results are mixed for C16, as two studies found an increased risk for CVD [32,33], two studies found no significant association [22,31], and the last reported significant heterogeneity [30].

Two studies found that a higher risk for CVD was associated with C18 intake [32,33], but no significant association was seen in two other studies after adjusting for confounding variables [22,31]. One study reported significant heterogeneity with pooled results [30]. Three studies looked at combined results for C12 through C18, two found a higher risk for CVD [22,33], and one study reported significant heterogeneity [30].

### 3.5. Food Sources

Four studies reviewed food sources and risk for CVD [22,30,31,32]. Most of the saturated fat came from cheese, milk/milk products, meat, fats (hard/solid), and butter [31,32]. Hu et al. reported the main sources of SFA were red meat and high-fat dairy products, which were also linked to an increased risk for CVD [22]. Conversely, low-fat dairy, poultry, and fish were linked to a lower risk [22]. Another study reported saturated fat intake from butter (HR: 0.94; 95% CI: 0.90, 0.99), milk (HR: 0.92; 95% CI: 0.86, 0.97), and cheese (HR: 0.91; 95% CI: 0.86, 0.97) had a significant reduction on risk for IHD (no significance from other foods) [31]. Cheese intake had a stronger impact on lowering risk of IHD in women (HR:0.89; 95% CI: 0.83, 0.96) than in men (HR: 0.97; 95% CI: 0.88, 1,07); no significance was found regarding sex otherwise [31]. In another study that evaluated two cohorts, one (EPIC-Norfolk) found no statistical significance with meat, dairy, cheese, soft/hard fats, or cookies and cakes and for isocaloric substitutions [30]. However, the EPIC-Denmark cohort indicated a higher risk of MI linked to a higher intake of SFA from meat (HR1en%: 1.08, 95% CI: 1.04, 1.12) [30]. The last study found no significant link with CVD and specific food groups (*p* > 0.46) [32].

### 3.6. Substitution

All the studies evaluated the impact of replacing calories from SFA with PUFA and risk for CVD [22,30,31,32,33]. Two studies found either no association [30] or the association was not statistically significant [32]. One study found mixed results, observing a significant reduction in the risk for CVD with isocaloric replacement of C16 with PUFA (HR1en%: 0.88; 95% CI: 0.81, 0.96; *p* = 0.002) [33]. However, no significant impact was observed with replacement of C18 with PUFA, even though there was a trend toward lower CVD risk (HR1en%: 92; 95% CI: 0.84, 1.01; *p* = 0.07) [33]. Conversely, isocaloric replacement of C12-18 combined with PUFA produced a reduced risk for CVD (HR1en%: 0.92; 95% CI: 0.89, 0.96; *p* < 0.001) [33]. Hu et al. found substitution with PUFA to have a strong inverse (lower risk) association with development of CVD [22]. Finally, the last study discovered a significantly higher risk of IHD when substituting SFA with PUFA (HR5en%: 1.35; 95% CI: 1.14, 1.61) [31]. Additionally, isocaloric replacement of C12-C18 combined with MUFA was found to have a reduced risk for CVD (HR1en%: 0.95; 95% CI: 0.90, 1.01; *p* = 0.08) [33]. However, another study found replacement with cis MUFA to have a higher risk of IHD (HR5en%: 1.30; 95% CI: 1.02,1.65) [31]. No significant impact was found in the remaining two studies that evaluated MUFA [30,32].

Four studies measured replacement of SFA with CHO [30,31,32,33]. One study found a significantly higher risk of IHD when SFA were replaced with CHO (HR5en%: 1.23; 95% CI: 1.09, 1.40) [31]. In addition, the type of CHO substitution made a big difference. Specifically, high-glycemic-index CHO significantly increased risk for IHD (HRGI > 56: 1.27; 95% CI: 1.03, 1.56), whereas low-glycemic-index CHO had no significant findings (HRGI > 56: 1.14; 95% CI: 0.91, 1.43) [31]. Zong et al. found a significant reduction in the risk for CVD with isocaloric replacement using whole-grain CHO (HR1en%: 0.90; 95% CI: 0.83, 0.97; *p* = 0.01) [33]. The last two studies either found no association at all [30] or no statistically significant results when substituting energy intake from SFA with CHO; this included accounting for quality of CHO [32].

Substitution with protein sources were measured as well [30,31,32,33]. Two studies reported that replacing SFA with animal protein caused a significantly higher risk of IHD (HR5en%: 1.37; 95% CI: 1.14, 1.65 [31]; HR5en%: 1.24; 95% CI: 1.01, 1.51 [32]). Four studies evaluated the use of plant protein for isocaloric replacement of SFA [30,31,32,33]. They found either a significant reduction in risk for CVD (HR1en%: 0.89; 95% CI: 0.82, 0.97; *p* = 0.01) [33], an inverse relationship (EPIC-Denmark cohort found substituting the sum of C12 and 14, C16, C18, and C12-C18 with plant protein had a reduced risk for MI, but this was not significant for the sum of C12 and C14) [30], or a neutral effect (HR5en%: 0.81; 95% CI: 0.57, 1.173 [31] HR5en%: 0.88; 95% CI: 0.50, 1.53 [33]).

### 3.7. Additional Analysis

Three of the studies in this review only obtained FFQ at the start of the cohort study [30,31,32]. Therefore, additional sensitivity analysis was completed to evaluate if large gaps in time was a limitation [30,31,32]. One measure eliminated the first two years, but this did not change the results [30,31,32]. Another measure conducted a sensitivity reanalysis at year 5 or 8 (depending on the study) to see if the shortened time would change the results, for which, two of the studies demonstrated no change [30,31]. In the last study, the 8-year investigation showed a statistically significant risk for CVD related to intake of C16 (HRSD: 1.62; 95% CI: 1.23, 2.15) and C18 (HRSD: 1.22; 95% CI:1.01, 1.47), a significant increased risk from meat (16%), and with substitution of saturated fat with cis MUFA (HR5en%: 1.54; 95% CI: 1.14, 2.07) [32]. In addition, there appeared to be a lower risk of CVD with SFA sources from cookies and cakes. This connection with cis MUFA and cookies and cakes were not seen in the results. The difference between the results and these may reflect changes in eating behavior that was not captured with a longer study period [32].

## 4. Discussion

### 4.1. Chain Length

In general, most studies found that LCSFA (C12-18) may increase the risk for CVD, while SCFA and MCFA (C4-C10) may be more beneficial or neutral [22,30,31,32,33]. However, researchers reported difficulty distinguishing between individual SFA because most food sources contain multiple types of saturated fatty acids [22,30,31,32,33]. A few studies assessed odd chain fatty acids as beneficial or neutral as well [30,31,32]. However, one study reported no association with LCSFA and CVD, with a slight benefit to consuming MCFA and odd chain SFA [31]. The consumption of C4-C10, C15, and C17 was relatively small as compared to C16 and C18 for all studies included in this review [22,30,31,32,33]. The low percentage of intake for C4-C10 may be to blame, in part, for the conflicting findings associated with CVD [22,30,31,32,33].

The research is inconclusive. However, similar to the studies reviewed in this paper, a recent meta-analysis including 12 studies reported a beneficial effect of coconut milk and coconut oil on lipid profile and CVD risk. When compared with plant oils and animal oils, coconut oil was found to significantly increase high-density lipoprotein cholesterol (HDL-C) by 0.57 mg/dL and 0.33 mg/dL, respectively. Coconut oil significantly raised low-density lipoprotein cholesterol (LDL-C) by 0.26 mg/dL compared with plant oils and lowered LDL-C compared with animal oils. No significant effects on triglyceride were observed. Better lipid profiles were demonstrated with the virgin form of coconut oil [34]. Because coconuts are not consumed as the only food source nor are a source of saturated fat, there is a need to assess overall dietary patterns when examining diet and CVD relationships [35,36].

The majority of SFA typically consumed by subjects participating in studies examining the connection between saturated fat and CVD was from C16 and C18 for meat and cheese [22,31,32]. In one study, C18 was the only SFA that demonstrated an increased risk for CVD development [32]. The main source of C18 was high fat/processed meat; therefore, it could be said that consumption of high fat/processed meat is a higher risk activity for the development of CVD than other sources [32,36]. Inconsistencies in these findings are likely impacted by shared food sources for SFA [22,30,32,33]. This makes it challenging to differentiate the health impacts of the different chain lengths and creates significant heterogeneity across studies related to chain length analysis. This, coupled with the low intake of C4-C10, makes it difficult to draw definitive conclusions regarding SFA chain lengths with regards to CVD risk.

### 4.2. Substitutions

Replacement macronutrients for SFA may play a larger role in the development of CVD than SFA itself [5,7,16,31]. In other words, if one decreases saturated fat intake, what does one replace it with? When it comes to PUFA, Hu et al. found the ratio of PUFA to SFA had a strong inverse link to the development of CVD, therefore suggesting LCSFA be replaced with PUFA [22]. This is consistent with findings from other studies and is generally an accepted practice for reducing CVD risk [2,14,37,38]. A recent systematic review about this topic concluded replacement of SFA with PUFA or MUFA to be beneficial [39]. However, one study included in this review did not find a beneficial effect, but rather a negative effect. The range of SFA intake was very narrow in this study (IQR: 13.2–16.6% of energy), which may have caused replacement modeling to be difficult to accurately capture. In addition, there was a high use of margarine (high in TFA) as the main source of PUFA [31]. The negative impact of TFA on CVD is well documented [17,31,37,40]. As a result, the researchers for this study did state these findings should be vigilantly scrutinized [31]. Another study included in this review did not find a significant association with replacement of SFA with PUFA or MUFA, but also noted the SFA intake range as a potential factor impacting their results [32]. A review by Chowdhury et al. likewise reported not finding an association with PUFA and MUFA with CHD risk [18]. However, this study has some inconsistencies in its statistical analysis that bring into question the weight of these findings [39]. The amount and type of SFA in relation to PUFA may be key in achieving beneficial results [13]. The evidence for MUFA has been inconclusive and does warrant further investigation [37]. Overall, findings for substitution with PUFA and MUFA were mixed, but use of PUFA seems to be better supported when limiting factors are taken into consideration.

Substitution results were also variable when SFA was substituted with CHO because the type of CHO consumed was not consistent between the studies [30,31,32,33]. Many studies found a higher intake of refined CHO with a lower fat intake to be more harmful than a higher fat intake consisting of unsaturated fat sources [19,38,41]. This may be related to the impact CHO have on blood lipid levels [19]. Carbohydrates can lower LDL but can also lower HDL and are linked to a higher total HDL ratio, triglyceride, and ApoB to ApoA1 ratio [19]. The type and quality of the CHO source seems to make a difference. Studies have found replacing SFA with whole grains instead of refined CHO has a beneficial impact on CVD risk [38,42]. In free living dietary intake, replacement CHO sources are often of low quality, added sugar or refined grains, having an associated increase in CVD risk [20,38]. This is evident by the fact that the primary source of calories in the U.S. diet are sugar and refined starch [33]. Therefore, from a practical perspective, substituting SFA with PUFA is more beneficial than replacing SFA with carbohydrates, especially refined carbohydrates [13,17].

As lower CHO diets continue to gain popularity, higher protein intake has become more prevalent [43]. Two of the studies in this review found using animal protein to replace SFA increases the risk for CVD [31,32]. Several other studies have also linked consumption of saturated fat from meat and processed meat as having high risk [7,14,36,44,45]. This may be related to the saturated fat, but it could also be other components of meat itself that collectively increase risk for CVD, such as high sodium and L-carnitine [44,46]. The link between meat intake and CVD development is observed more significantly in U.S. cohort studies due to higher consumption of red and processed meats in the USA [44]. Furthermore, four of the studies found replacing SFA with plant protein to be of benefit [30,31,32,33]. Other studies have found similar results with increased intake of plant-based protein [46,47]. However, this connection with protein sources is not consistent [43]. Two recent meta-analyses found only a weak association at best [48,49]. Veernooij et al. examined 70 cohort studies, covering over six million participants, looking at diet patterns associated with meat consumption, finding low evidence that eating less red or processed meat reduced the risk of CVD. Significant heterogeneity was noted across the cohorts however, which likely influenced these results [48]. Likewise, Zeraatkar et al. found a similar association but also noted limitations to adequately control confounding variables [49].

Findings for dairy products in relation to CVD risk remain unclear as well. The studies in this review either found a slight benefit or no relationship [22,30,31,32]. Other studies have found a possible link with the SFA from dairy and CVD [9,18,50,51]. Chen et al. assessed dairy fat intake compared with other fats in three cohorts over a four-year period, concluding no association with risk for CVD. In fact, isocaloric replacement of dairy fat with animal fat resulted in a significant increase in CVD risk [50]. However, isocaloric replacement of dairy fat with PUFA, vegetable fat, and complex CHO resulted in a significantly lower risk of CVD [50]. Dairy fat is mostly LCSFA (C12-18) [50], while animal fat is primarily C18 [31,32]. Mazidi et al. recently conducted a cohort study/meta-analysis looking at the link between dairy itself and all-cause mortality. One of the things evaluated in this analysis was the heterogeneity of this food category and its nutrition properties, suggesting different sources of dairy foods have different health impacts. These researchers found a higher risk of mortality of CHD with increased milk consumption, but a lower risk was seen with consumption of fermented dairy products [51]. It has been suggested that the production of SCFA from microbial fermentation may explain the beneficial outcomes [51,52]. Conflicting findings between the SFA profile of dairy products vs. individual SFAs may be explained by another nutrient component in dairy, such as calcium [8,9,31]. Overall, findings with dairy products were more positive than not, but still not strong enough to make a definitive conclusion.

Differences in the overall diet may explain some of the variances between the findings in this systematic review. Specifically, in the USA, the major source of SFA is meat/meat products, whereas in Europe, the main source is from dairy, while in other countries that were not included in these studies, the source is coconut and other plant-derived SFA sources [30]. The population characteristics in these cohort studies may also be an uncontrolled variable [19]. Often, these cohort studies tend to be in countries with higher economic status, with less extremes in nutritional intake among the population [19]. Additionally, the difference in findings between the USA and European cohort could be related to repeat FFQ follow ups in the USA that likely resulted in more accurate data [30]. Researchers attempted to control for this in the European cohorts by conducting sensitivity analyses of shortened follow-up times [30,31,32]. In one study, the first 8 years had different results compared with the end analysis. The difference between the final and earlier results may reflect changes in eating behavior that were not captured in a longer study period with no re-evaluation of dietary patterns [32].

### 4.3. Limitations

There are limitations to the studies included in this review. First, cohort studies are inherently biased by design due to the reliance on self-reported data [3,29]. Even though the FFQs used were previously validated and then cross-verified with food records or 24 h recalls, they still ultimately rely on self-reporting and therefore can contribute to bias [3,31,32,33,35]. In addition, the FFQ used in the EPIC-Norfolk cohort was not validated for measuring saturated fat, and the Danish cohort had limited information on TFA or C17 intake [30]. All three European studies only obtained dietary data at initiation of the study, without reassessing for changes [30,31,32]. Future studies should consider adding assessment of blood lipids in a small subgroup of the cohort to validate data obtained from 24 h recall [53]. Another limitation related to lack of follow-up in the European studies is the possibility of a participant being started on a cholesterol lowering medication or other treatment modalities that could have impacted the findings [31]. Likewise, whether screening was conducted for the development of co-morbidities that may increase risk for CVD during the course of the study would be an added limitation.

When it came to analyzing individual SFA, there was a lot of difficulty distinguishing between individual SFAs due to the shared food sources [22,33]. In addition, some of the SFA were consumed in very small amounts, making the ability to conclude risk difficult [17]. Both issues make the practicality of measuring individual SFA questionable [22,33]. This review identified the whole food source, or better yet, dietary patterns, as possibly being more important when minimizing risk of CVD with diet [17,32]. Other components of whole foods may play a vital role and are not captured when isolating an individual nutrient.

Finally, even though all studies were adjusted for multiple confounding factors, it is possible that some residual confounding factors could have skewed results [17,31,33]. For example, the use of health professionals in the NHS cohort with similar socioeconomic status and ethnic background could limit generalizability of the findings [33]. Similarly, the older population of the Rotterdam cohort could lead to different dietary patterns not universally applied [31,32].

## 5. Conclusions

Overall, the results of this systematic review on SFA were inconclusive. However, there is compelling evidence to focus further research on dietary intake of different types of saturated fats and CVD risk [36]. The focus on saturated fat is based on its impact on LDL, but perhaps dietary influence on other lipoproteins and risk factors for CVD should be considered [19]. Saturated fat intake cannot be considered as an isolated dietary component that might exert health benefits when intake is reduced; rather, dietary patterns should be closely examined, such as the Mediterranean diet or vegetarian diets. For example, in this review, nearly all participants who consumed the highest amounts of saturated fat also consumed a low-fiber diet [22,30,31,32]. Research has demonstrated that fiber can help reduce the risk for CVD, and typically a high diet high in saturated fat is low in fiber [26,36,41]. It may be best to change the emphasis from nutrient- or macronutrient-specific recommendations to whole-food- or diet-pattern-specific recommendations [17,20,52]. Additionally, consideration should be given for total lifestyle choices as all-encompassing, not only diet or one specific component of diet [36,54]. The studies in this review found that people who consume a higher saturated fat diet tend to also smoke, are less active, have higher BMIs, eat less fiber, etc., which can also contribute equally to increased CVD risk. Furthermore, grouping all saturated fats into one category and therefore one recommendation is remiss, as they clearly have different health impacts based on their varying fatty acid chain lengths. The studies included in this review suggest that LCSFA (C12-18) may increase the risk for CVD development, while SCFA and MCFA (C4-C10) may be more beneficial or neutral [22,30,31,32,33]. Clearly, more research is needed to assess the impact of the different types of saturated fat on CVD and other health outcomes.

## Figures and Tables

**Table 1:** Bias assessment.

Study					NIH Criteria
Hu et al., 1999 [22]	Praagman et al., 2016 [32]	Praagman et al., 2016 [31]	Zong et al., 2016 [33]	Praagman et al., 2019 [30]	
Yes	Yes	Yes	Yes	Yes	(1) Clear objective research questions
Yes	Yes	Yes	Yes	Yes	(2) Defined study population
Yes	Yes	Yes	Yes	Yes	(3) Participant of >50% of eligible persons
Yes	Yes	Yes	Yes	No	(4) Consistent subject selection/recreation and predetermined exclusion/inclusion criteria applied
NR/UTD	NR/UTD	NR/UTD	NR/UTD	NR/UTD	(5) Sample size justification/stats provided
Yes	Yes	Yes	Yes	Yes	(6) Exposures measured prior to outcome
Yes	Yes	Yes	Yes	Yes	(7) Appropriate timeframe to assess desired exposure and outcome
Yes	Yes	Yes	Yes	Yes	(8) For variable exposures, different levels were assessed
Yes	Yes	Yes	Yes	Yes	(9) Clearly defined independent variable consistently implemented
Yes	No	No	Yes	No	(10) Repeat measure of exposure over time
Yes	Yes	Yes	Yes	No	(11) Clearly defined dependent variables consistently implemented
Yes	NR/UTD	NR/UTD	Yes	Yes	(12) Blinded outcome assessors
NR/UTD	NR/UTD	NR/UTD	NR/UTD	No	(13) Loss to follow up < 20%
Yes	Yes	Yes	Yes	No	(14) Confounding variables identified and adjusted statistically

Table created using Study Quality Assessment Tools | NHLBI, NIH. https://www.ncbi.nlm.nih.gov/pubmed/NR/UTD (Accessed on 12 October 2022), not reported or unable to determine.

**Table 2:** Study characteristics.

Study (Year Published)	Praagman et al. (2016) [31]	Zong et al. (2016) [33]	Praagman et al. (2019) [30]	Hu et al. (1999) [22]	Praagman et al. (2016) [32]
Cohort (country)	EPIC-NL (the Netherlands) MORGEN (the Netherlands)	NHI (USA) HPFS (USA)	EPIC-Norfolk (the Netherlands) EPIC-Denmark (the Netherlands)	NHS (USA)	Rotterdam (the Netherlands)
Number of Participants in Cohort (gender)	17,357 EPIC-NL (women) 22,645 MORGEN (men/women)	121,700 NHS (women) 51,529 HPFS (men)	25,639 EPIC-Norfolk (women/men) 57,053 EPIC-Denmark (women/men)	121,700 (women)	7983 (women/men)
Age at baseline (years)	40–70 (EPIC-NL) 20–65 (MORGEN)	35–55 (NHS) 40–75 (HPFS)	40–74 (EPIC-Norfolk) 50–64 (EPIC-Denmark)	33–55	≤55
Exclusions	Subjects that denied access to vitals/death records; missing questionnaires; implausible energy intake; CVD at baseline	Diagnosis of cancer, DM, or CVD at baseline; implausible energy intake or missing info; incomplete questionnaire or missing age at baseline	History of cancer, CVD, MI, incomplete diet data, implausible intake, missing data re: co-variables	If greater than 10 items left blank on FFQ, implausible intake, diagnosis cancer, angina, MI, stroke, DM, high cholesterol, or other CVD	Institutionalized participants, subjects who had difficulty “recalling” diet patterns or suspected to have dementia, no informed consent for diet analysis, lost to follow-up, prevalent CVD
Number of participants included in the study	35,597	115,782	74,425	80,082	4722
Mean duration of follow-up (years)	12.2	25.8 (women) 21.2 (men)	EPIC-Norfolk: 18.8 EPIC-Denmark: 13.6	14	16.3
Measure of dietary assessment	FFQ	FFQ	FFQ	FFQ	FFQ
Validation of dietary assessment	Yes, 24 h recall	Yes, 24 h recall	NS	Food records	Food records
Assessment of SFA chain length	Dutch Food Composition Table 1998	USDA and Harvard University food composition database	McCance and Widdowson’s The Composition of Food	U.S. Department of Agriculture	Dutch Food Composition Table 1998
Ascertainment of disease development	Dutch Center for Health Care Information (EMR)	Self-report, next of kin/national death records; then confirmed by study physicians, EMR	Patient registry, death certificates, EMR	Study physicians confirmed diagnosis (blinded); deaths vs. national death index or next of kin, hospital records	EMR, study physicians
Other variable adjustments	Smoking status, education, physical activity, anthropometrics, BP, total cholesterol concentration, alcohol intake.	Age, ethnicity, family history of MI, BMI, smoking status, alcohol status, physical activity, use of MVI, menopause/post-menopausal and/or hormone use, HTN, aspirin use, cholesterol status, total energy intake.	Measure for food sources and SFA content as well (meal/dairy vs. cake/cookies, cheese, hard fat and MI). Other variables controlled for: medication use, alcohol use, smoking, physical activity, ht/wt and waste circumference, HTN status, BMI, menopausal/post-menopausal, hormone replacement, high cholesterol.	BMI, smoking, menopause/postmenopausal, hormone replacement, history of MI in family, MVI, vitamin E supplements, alcohol.	Body weight, weight circumference, height, blood pressure, BMI, smoking status, education, household income, serum cholesterol levels, physical activity.

EPIC-NL, European Prospective Investigation into Cancer and Nutrition-Netherlands; MORGEN, Monitoring Project on Risk Factors for Chronic Diseases; CVD, cardiovascular disease; FFQ, food frequency questionnaire; EMR, electronic medical record; BP, blood pressure; NHI, Nurses’ Health Study; HPFS, Health Professionals Follow-up Study, DM, diabetes mellitus; MI, myocardial infraction; BMI, body mass index; MVI, multivitamin; HTN, hypertension; NS, not stated; SFA, saturated fatty acids.
